# DNA methylation profile in beef cattle is influenced by additive genetics and age

**DOI:** 10.1038/s41598-022-16350-9

**Published:** 2022-07-14

**Authors:** André Mauric F. Ribeiro, Leticia P. Sanglard, Hiruni R. Wijesena, Daniel C. Ciobanu, Steve Horvath, Matthew L. Spangler

**Affiliations:** 1 Department of Animal Science, University of Nebraska, Lincoln, NE 68583 USA; 2 Department of Human Genetics, David Geffen School of Medicine, University of California, Los Angeles, CA 90095 USA; 3 Department of Biostatistics, Fielding School of Public Health, University of California, Los Angeles, CA 90095 USA

**Keywords:** Genetics, Biomarkers

## Abstract

DNA methylation (DNAm) has been considered a promising indicator of biological age in mammals and could be useful to increase the accuracy of phenotypic prediction in livestock. The objectives of this study were to estimate the heritability and age effects of site-specific DNAm (DNAm level) and cumulative DNAm across all sites (DNAm load) in beef cattle. Blood samples were collected from cows ranging from 217 to 3,192 days (0.6 to 8.7 years) of age (*n* = 136). All animals were genotyped, and DNAm was obtained using the Infinium array HorvathMammalMethylChip40. Genetic parameters for DNAm were obtained from an animal model based on the genomic relationship matrix, including the fixed effects of age and breed composition. Heritability estimates of DNAm levels ranged from 0.18 to 0.72, with a similar average across all regions and chromosomes. Heritability estimate of DNAm load was 0.45. The average age effect on DNAm level varied among genomic regions. The DNAm level across the genome increased with age in the promoter and 5′ UTR and decreased in the exonic, intronic, 3′ UTR, and intergenic regions. In addition, DNAm level increased with age in regions enriched in CpG and decreased in regions deficient in CpG. Results suggest DNAm profiles are influenced by both genetics and the environmental effect of age in beef cattle.

## Introduction

DNA methylation (DNAm) is an epigenetic mark characterized by a modification of the chromatin structure in mammals^1^. This modification exists throughout the genome with remarkably less abundance in cytosine-guanine (CpG) islands^2^. In mammalian genomes, CpG islands are greatly under-represented due to spontaneous evolutionary deamination of 5-methylcytosine to thymine and are usually located within or near promoters and first exons of housekeeping genes^3^. In general, it has been recognized that DNAm in the promoter region of genes indicates transcriptional suppression while hypomethylation is associated with transcriptional activation and increased gene expression^2^.

DNA methylation profiles are dynamic and susceptible to factors such as age^4–6^, diet^7,8^, sex^9^, and exposure to diseases^10^. It has been established that the DNAm level of mammalian genomes increases with age, although this relationship seems to differ across the regions of the genome. Marttila et al.^11^ observed that age-associated DNA hypermethylation seems to originate from programmed developmental changes while DNA hypomethylation could result from environmental influences^11^. Furthermore, DNAm profiles in several species have been demonstrated to predict individuals’ biological age, known as epigenetic clocks^12–23^. Recently, Caulton et al.^13^ reported a prediction accuracy of 0.95 for epigenetic clocks based on DNAm in a dairy herd. In general, DNAm has been considered a promising indicator of biological age in mammals.

In addition to influences of environmental factors, genetic control of DNAm levels has been reported^9,24,25^. For example, DNAm of human peripheral blood lymphocytes at sites with deficient CpG density tended to have higher heritability than regions with high-CpG density^25^. Integrating genomic data with DNAm information could be a potential strategy to increase the accuracy of phenotypic prediction in livestock. However, the expected improvement depends on the heritability of DNAm, or in other words, the additional information beyond additive genetics to be gained with DNAm information. Therefore, the objectives of this study were to estimate the heritability and age effects of site-specific and cumulative DNAm across the cattle genome.

## Results

### Description of DNAm level and load

**Figure 1:**
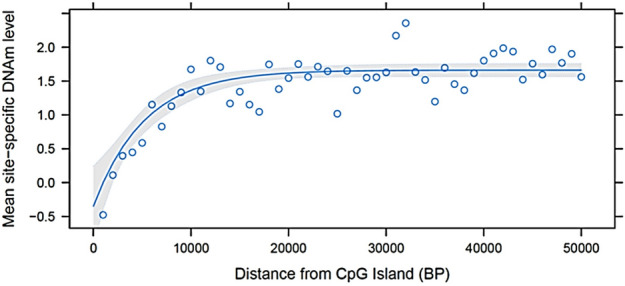
Exponential regression of mean DNAm level on distance of the methylated cytosines from CpG islands into bins of 1000 bp.

DNA methylation levels, defined as logit transformed standardized intensity (M-value), varied with functional regions of the genome (Table 1). On average, promoter (M-value =  − 1.28) and 5′ UTR (M-value =  − 1.31) were hypomethylated while exonic, intronic, 3′ UTR, and intergenic regions were hypermethylated, with M-values ranging from 0.83 to 1.24. CpG islands were hypomethylated (M-value =  − 0.68), while regions deficient in CpG were hypermethylated (M-value = 1.18). The average DNAm load, defined as the sum of M-values across all DNAm sites, was 12,336 ± 3,584. The animals with the highest and lowest DNAm load had 20,808 and 6,399 sites, respectively. Additionally, as the distance of methylated cytosines from the CpG islands increased, there was an exponential increase in the mean site-specific DNAm level, reaching a plateau when the distance was approximately 1 kb (Fig. 1).

**Table 1:** Summary of estimated parameters for DNA methylation (DNAm) levels (M-values) for all DNAm sites in different functional regions of the bovine genome.

Parameters		Promoter	5′ UTR	Exon	Intron	3′ UTR	Intergenic	All Regions
Number of sites		2,445	1,456	11,500	7,852	879	10,192	34,324
M-value	Mean ± SD	− 1.28 ± 1.97	− 1.31 ± 2.36	1.24 ± 2.20	1.22 ± 2.08	1.63 ± 1.93	0.83 ± 2.01	0.84 ± 2.24
	Maximum	5.67	5.27	5.57	5.49	4.90	5.08	5.67
	Minimum	− 5.25	− 5.27	− 5.51	− 5.18	− 4.83	− 5.38	− 5.51
${\sigma }_{p}^{2}$	Mean ± SD	0.05 ± 0.16	0.04 ± 0.05	0.04 ± 0.11	0.05 ± 0.15	0.04 ± 0.05	0.06 ± 0.23	0.05 ± 0.17
	Maximum	6.24	0.75	4.93	5.18	2.73	6.51	6.51
	Minimum	0.00	0.00	0.00	0.00	0.00	0.00	0.00
${\sigma }_{a}^{2}$	Mean ± SD	0.02 ± 0.09	0.01 ± 0.02	0.02 ± 0.06	0.02 ± 0.08	0.02 ± 0.07	0.03 ± 0.14	0.02 ± 0.10
	Maximum	3.33	0.33	2.97	2.85	1.62	4.08	4.08
	Minimum	0.00	0.00	0.00	0.00	0.00	0.00	0.00
${h}^{2}$	Mean ± SD	0.36 ± 0.07	0.35 ± 0.07	0.34 ± 0.07	0.34 ± 0.07	0.34 ± 0.07	0.36 ± 0.08	0.35 ± 0.07
	Maximum	0.67	0.63	0.69	0.71	0.72	0.73	0.73
	Minimum	0.19	0.20	0.18	0.20	0.20	0.19	0.18
Age effect	Mean ± SD	9.2E − 05 ± 1.6E − 04	4.8E − 05 ± 1.2E − 04	− 3.8E − 05 ± 1.3E − 04	− 5.4E − 05 ± 1.4E − 04	− 5.7E − 05 ± 1.4E − 04	− 6.8E − 05 ± 1.6E − 04	− 3.8E − 05 ± 1.6E − 04
	Maximum	9.9E − 04	1.0E − 03	1.1E − 03	7.0E − 04	7.7E − 04	8.9E − 04	1.1E − 03
	Minimum	− 7.6E − 04	− 5.2E − 04	− 7.1E − 04	− 5.6E − 04	− 4.8E − 04	− 8.6E − 04	− 8.6E − 04

${\sigma }_{p}^{2}$, phenotypic variance (i.e., additive genetic variance + residual variane); ${\sigma }_{a}^{2}$, additive genetic variance; ${h}^{2}$, heritability.

### Genetic parameters of DNAm level and load

**Figure 2:**
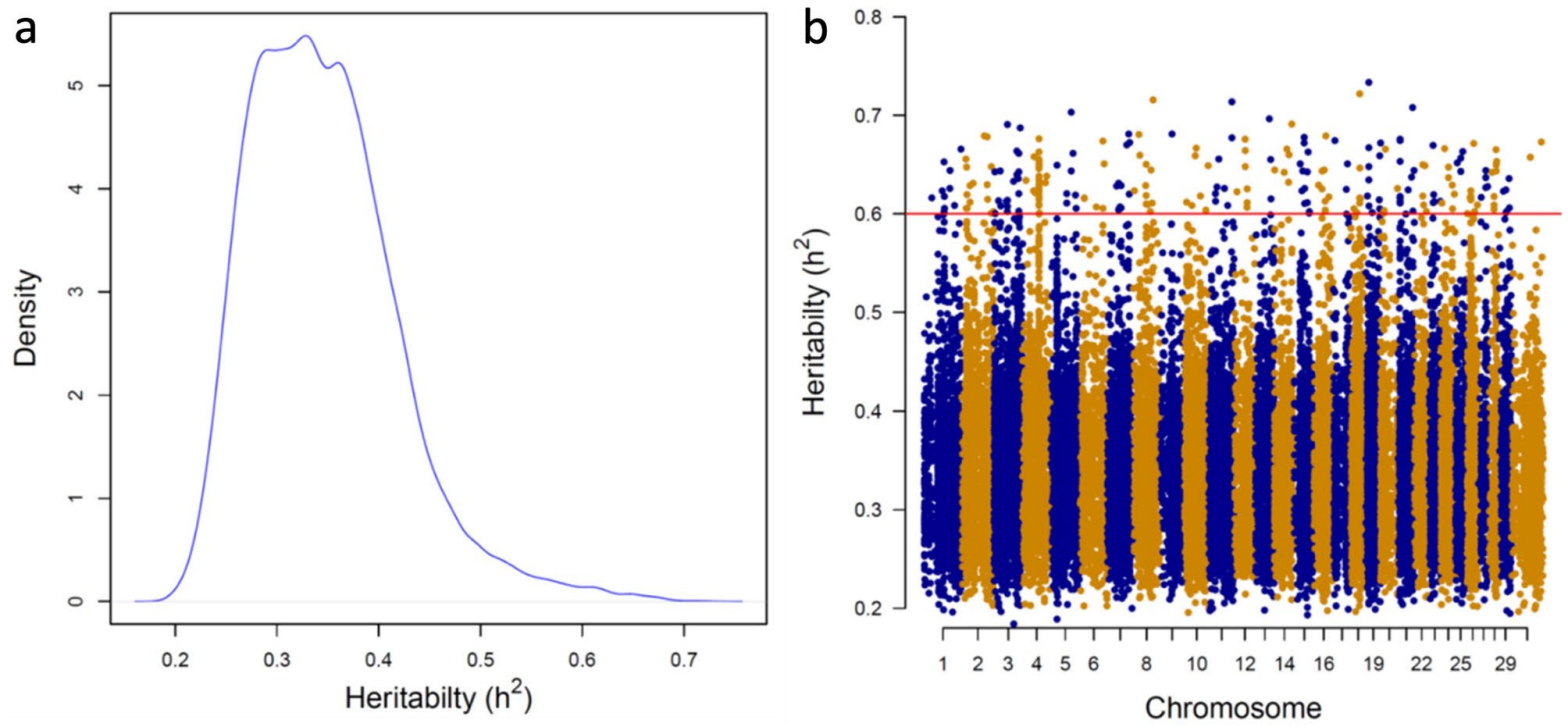
Density of heritability estimates of DNAm levels for each DNAm site (**a**) and heritability estimates of DNAm levels across all chromosomes (**b**).

Estimates of heritability (h^2^) of DNAm sites ranged from 0.18 to 0.72 (Fig. 2a). A similar average estimate was obtained across the functional regions, ranging from 0.34 ± 0.07 to 0.36 ± 0.08 (Table 1). The distribution of h^2^ estimates was uniform across the genome (Fig. 2b). The distribution of genetic and residual variance estimates across the genome is depicted in Supplemental Fig. S1. Chromosomes 20 and 21 showed the smallest and largest average heritabilities (0.35 ± 0.08 and 0.37 ± 0.08), respectively. The h^2^ estimate of DNAm load was moderate to high (0.45 ± 0.10). The average h^2^ estimates of DNAm were similar in regions enriched or deficient in CpG islands (0.36 ± 0.07 and 0.36 ± 0.08, respectively).

### Age effect on DNAm level and load

**Figure 3:**
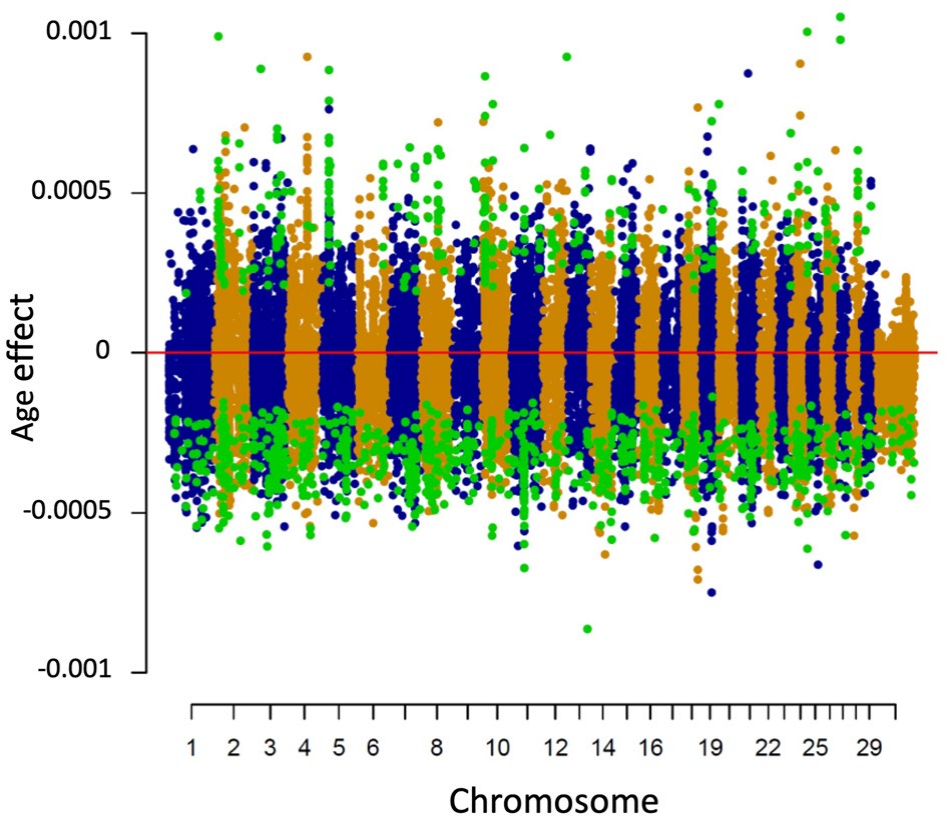
Distribution of age effects on DNAm levels across all chromosomes. DNAm sites with significant age effect (*P* ≤ 10^–25^) are highlighted in green.

Overall, the average age effect on DNAm level across all sites was negative (-0.013 ± 0.06) (Fig. 3); however, the effect of age varied by functional region. The DNAm level increased with age in the promoter and 5′ UTR and decreased in the exonic, intronic, 3′ UTR, and intergenic regions (Table 1). In addition, DNAm level increased with age in regions enriched in CpG islands and decreased in regions deficient in CpG islands (Results not shown). DNAm sites with significant age effects were observed when h^2^ estimates were intermediate rather than extreme values (Supplemental Fig. S2).

In addition, DNAm level increased with age in regions enriched in CpG islands and decreased in regions deficient in CpG islands (Results not shown). DNAm sites with significant age effects were observed when h^2^ estimates were intermediate rather than extreme values (Supplemental Fig. S2).

**Figure 4:**
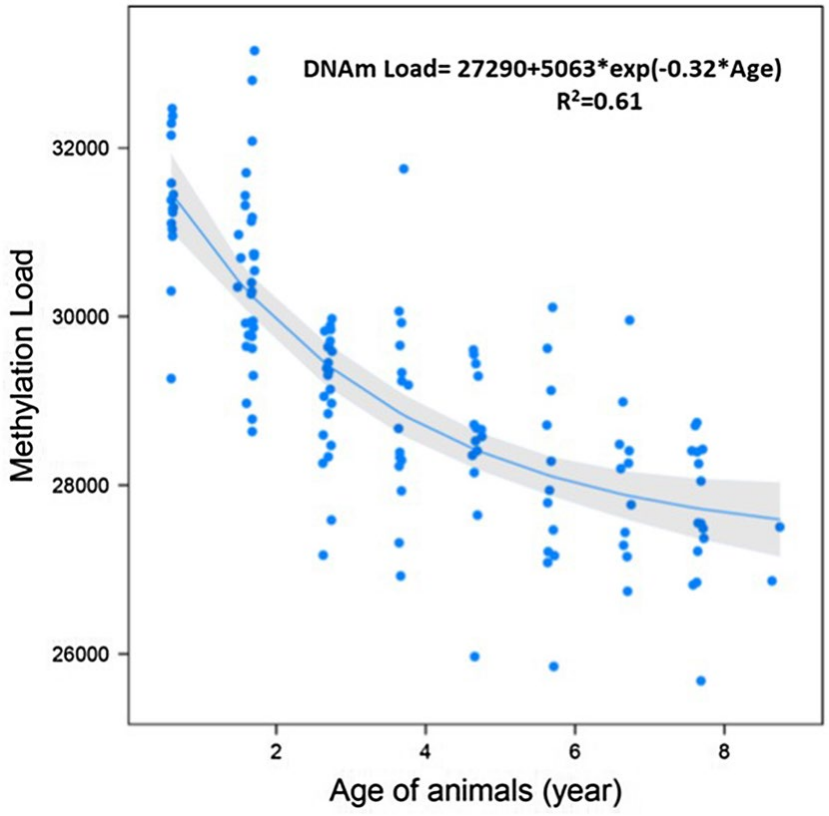
Exponential decay regression of Methylation load (DNAm load) on the age of animals (years).

**Figure 5:**
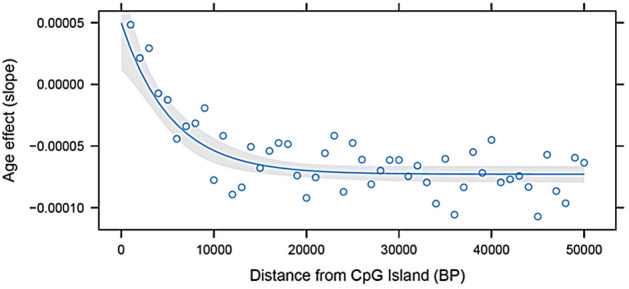
Exponential regression of age effect on distance of the methylated cytosines from CpG islands into bins of 1000 bp.

There was a non-linear relationship between DNAm load and animal age (years) (${R}^{2}=0.61$, Fig. 4), with younger animals having a greater DNAm load than older animals. As the distance from CpG islands increased, there was an exponential decrease in age effect on DNAm, reaching a plateau when the distance was approximately 1 kb (Fig. 5).

Predicted values for age were generated from five Bayesian regression models: Bayesian Ridge Regression (BRR), BayesA, BayesB, BayesCπ, and Bayesian LASSO (BLASSO). Results can be seen in Table 2. In general, the mean correlation (r) between the predicted and true ages was high, ranging from 0.97 to 0.99, and the mean slope of the regression of predicted on true age ranged from 0.88 to 0.92, close to the expected slope of 1. A BayesA model performed best (r = 0.99, MSE = 0.11 and slope = 0.93), while BLASSO was the least accurate, albeit similar, and yielded predictions that were under-dispersed to a greater degree (r = 0.97, MSE = 0.26 and slope = 0.88).

**Table 2:** Mean correlation, MSE, and slope of regression of predicted age based on DNAm status on true age.

Parameter	BRR	BayesA	BayesB	BayesCπ	BLASSO
Mean correlation	0.98 (0.003)	0.99 (0.003)	0.98 (0.003)	0.98 (0.003)	0.97 (0.006)
Mean of MSE	0.15 (0.036)	0.11 (0.031)	0.14 (0.033)	0.15 (0.035)	0.26 (0.071)
Mean of slope	0.92 (0.023)	0.93 (0.021)	0.92 (0.022)	0.92 (0.023)	0.88 (0.031)

*MSE* Mean Square Error; *BRR* Bayesian Ridge Regression; *BLASSO* Bayesian LASSO.

## Discussion

Changes in DNA methylation profiles can alter gene expression leading to phenotypic variation. DNA methylation is subjected to changes due to age and potentially by underlying genetic variation. Furthermore, methylation levels can vary across the genome depending on the functional role of the region and the location and enrichment of CpG islands. This study estimated heritabilities and the effect of age on site-specific and cumulative DNAm in a beef cattle population.

DNAm level was lower in the promoter and 5′ UTR than 3′ UTR, intergenic, exonic, and intronic regions. An exonic region was defined as a region of the genome that forms an mRNA molecule and is translated into a protein. Correspondingly, a lower DNAm level was observed in CpG islands than non-CpG islands. An increase in DNAm levels was observed as their distance from the CpG islands increased. This was expected given many promoters in the mammalian genome are characterized by unmethylated CpG islands, and promoter hypomethylation is associated with transcriptional activation^1^. In addition, hypomethylation in the 5′ UTR could be because CpG site-associated genes usually extend from the 5′ UTR end into the gene body^1^. Hypermethylation in intronic and exonic regions was also observed by Chodavarapu et al.^26^ and was associated with an enrichment of nucleosomes, which are the basic structural unit of DNA packaging in eukaryotes and correspond to segments of DNA coiled around a core of histone proteins. However, different from this study, the authors observed a greater DNAm level in exons than in introns^26^. The hypermethylation in coding exons and 3′UTR is consistent with the presence of DNAm in transcribed regions.

Heritability estimates of DNAm levels were similar and moderate among functional regions, CpG islands vs. non-CpG islands, and chromosomes. These results indicate potentially no relationship between h^2^ of DNAm levels and CpG density. These results contrast with McRae et al. and Gordon et al.^24,25^, who reported higher h^2^ of DNAm levels in low-CpG density sites than regions with high-CpG density in the genome of human peripheral blood lymphocytes. Estimates of heritability ranged from moderate to high across DNAm sites and were high for DNAm load, in agreement with previous reports. Boks et al.^9^ estimated h^2^ ranging from 0.03 to 0.90 in specific CpG islands in a twin study. Another twin study reported h^2^ estimates ranging from 0.48 to 0.94 for the top 5% of sites investigated in cord blood mononuclear cells^24^. These results indicate that a substantial part of the variation in DNAm is due to genetic variance. However, there is still extra information beyond additive genetics to be gained with DNAm patterns that could help improve phenotypic prediction, especially for lowly heritable traits.

The relationship between DNAm level and aging has been well established. In the current study, the effect of age was dependent on the genomic region. DNAm level increased with age in the promoter and 5' UTR but decreased in the other regions. Similarly, DNAm level increased with age in CpG regions but decreased in non-CpG regions. These results are in agreement with Marttila et al.^11^, who observed enrichment of aging-associated hypermethylation at CpG islands and hypomethylation at non-CpG islands. The majority of CpG islands are not initially methylated, and the change observed during aging is hypermethylation. The opposite is true for regions with few CpG islands that initially are heavily methylated, and the non-CpG are associated with hypomethylation. Marttila et al.^11^ observed a slight majority (54%) of the identified sites were hypomethylated in the elderly. Similarly, Johansson et al.^27^ also reported an excess of hypomethylation over hypermethylation with aging. On the other hand, Korkmaz and Kerr^28^ observed hypomethylation of 67% of the CpG islands analyzed in 5 m vs. 16 m old dermal fibroblasts in dairy cattle. Age-associated DNA hypermethylation seems to be associated with genes belonging to a common pathway of sequence-specific DNA binding and transcription factor binding regulating genes associated with development and morphogenesis; and metabolic processes, gene expression and nucleotide metabolism, while hypomethylation was not associated with specific pathways^11^.

The current study also showed that age could be accurately (r ≥ 0.97) predicted based on DNAm status. In general, all models slightly under-predicted age. The slightly better performance of BayesA indicated that fitting all DNAm in the model simultaneously improves age prediction, and there is no advantage in performing variable selection^29^. Additionally, BayesA assumes a heavy-tailed distribution of effects that may be more appropriate for modeling the effects of DNAm on age^29^. Previous studies have shown DNAm can be used as features for constructing epigenetic clocks in several species, including cattle. Caulton et al.^13^ reported a high prediction accuracy (> 0.94) for different age clocks based on DNAm of CpG islands in deer, goat, sheep, and dairy cattle. More recently, based on DNAm of liver and blood samples of horses, Horvath et al.^16^ reported accuracy greater than 0.95 for epigenetic clocks. Kordowitzki et al.^18^ developed epigenetic clocks of reproductive aging in oocytes and blood from cattle with an accuracy greater than 0.86. Hayes et al.^17^ achieved a prediction accuracy for birthdate of 0.71 in beef cattle from a diverse genetic background. These results are encouraging for using epigenetic clocks to estimate age in cattle reared in extensive production systems, which usually have unknown or inaccurate birthdates records. However, accurately predicting differences in birthdates among cohorts requires substantial reductions in the average error reported by Hayes et al. that could perhaps be achieved with much larger training sets. Overcoming the challenges of industry birthdate recording with epigenetic clocks could contribute to herd management and estimation of genomic breeding values for economically important traits that depend on accurate age records, such as growth rate and calving interval^17^. Given epigenetic clocks have proven to be accurate predictors of biological age, epigenetic indicators could provide a mechanism to infer age-related pathologies and identify genetic, environmental, or lifestyle factors that accelerate or slow aging in humans and livestock^30^.

## Material and methods

### Population resource and data collection

All animal procedures implemented in this study were approved by the University of Nebraska-Lincoln Animal Care and Use Committee. All methods were performed in accordance with relevant guidelines and regulations. Methods are reported in the manuscript following the recommendations in the ARRIVE guidelines.

The blood obtained from 136 cows was centrifuged at 2500 × g for 10 min at room temperature. The buffy coat was collected and stored at − 80 °C. All samples were collected from October 2 to November 27 of 2018 at the Eastern Nebraska Research and Extension Center at the University of Nebraska-Lincoln. Cows were chosen to provide variation in age and ranged from 217 to 3,192 days (0.6 to 8.7 years) of age at the time of sample collection. Animal were from an admixed population comprised of purebred Angus and composites of differing proportions of Angus, Simmental, and Red Angus.

All animals were genotyped with the medium-density Illumina BovineSNP50 (~ 50 K SNPs) BeadChip (Illumina, San Diego, CA, USA). Genotype filtering included removing non-autosomal SNPs, SNPs with minor allele frequency < 0.02, and SNPs with Hardy–Weinberg equilibrium *P*-value > 10^−5^.

### DNA methylation

DNA was extracted from buffy coats using the DNeasy Blood and Tissue Kit (Qiagen, Cat No. 69506) and, subsequently, bisulfite-converted using the EZ DNA Methylation Kit (ZymoResearch, Irvine, CA, USA). DNAm data was obtained from bisulfite-treated samples using the mammalian array (HorvathMammalMethylChip40)^31^. DNAm level for each site was calculated as methylation β-value (β-value = intensity of the methylated allele/intensity of the unmethylated allele + intensity of the methylated allele + 100). The addition of 100 was used to stabilize β-values when both intensities of the methylated and unmethylated alleles were small^32^. The SeSAMe pipeline^33^ was used to generate normalized β-values and for quality control. The β-value has severe heteroscedasticity outside the intermediate methylation range; thus, a logit transformation of the β-values (M-values) was used to approximate homoscedasticity. M-values of 0 correspond to 50% of methylation, and positive and negative values correspond to greater and lesser than 50% methylation level, respectively. M-values were used to quantify DNAm level by region (i.e., promoter, 5′ and 3′ UTR, exonic, intronic, and intergenic) and location related to CpG islands (i.e., within or outside). DNA methylation load was calculated as the sum of all DNAm levels (M-value).

DNAm status of each site was determined by the distribution of the methylation β-values. For example, β-values below, within, and above 2 standard deviations were classified as unmethylated (0), intermediately methylated (1), and methylated (2), respectively. DNAm status was used for prediction purposes.

### Statistical analyses

#### Genetic parameters for DNAm level and DNAm load

Genetic parameters (i.e., additive genetic and residual variances) for DNAm level (M-values) and DNAm load were estimated using the following animal model fitted in a Bayesian genomic best linear unbiased prediction (GBLUP) framework.

$${\varvec{y}}={\varvec{X}}{\varvec{b}}+{\varvec{Z}}{\varvec{u}}+{\varvec{e}}$$

where ${\varvec{y}}$ corresponds to the vector of phenotypes (DNAm level or DNAm load); ${\varvec{X}}$ corresponds to the design matrix linking the fixed effects to the phenotypes; ${\varvec{b}}$ corresponds to the vector of fixed effects including the intercept, the linear and quadratic (DNAm only) covariates of age, and covariates for proportion of each breed; ${\varvec{Z}}$ corresponds to the incidence matrix linking the random animal effect to the phenotypes; ${\varvec{u}}$ corresponds to the vector of random animal effects, where ${\varvec{u}}$ ~ N(0, **G** ${\sigma }_{u}^{2}$), where **G** corresponds to the the genomic relationship matrix constructed following the first method of VanRaden^34^ and ${\sigma }_{u}^{2}$ corresponds to the additive genetic variance; and ${\varvec{e}}$ corresponds to the vector of random residual effects associated with the phenotype, where ${\varvec{e}}$ ~ N(0, **I** ${\sigma }_{e}^{2}$), where I corresponds to the identity matrix and ${\sigma }_{e}^{2}$ corresponds to the residual variance. The h^2^ was obtained as the ratio of additive genetic variance divided by phenotypic variance (additive genetic variance + residual variance).

Gibbs sampling was used to sample a posterior parameter distribution with a chain length of 20,000 iterations, burn-in of 2,000 samples, and a thinning interval of 100. Analyses were performed using the *BGLR* package^35^ in R software.

#### Age effect on DNAm load and age prediction

The effect of age on DNAm load was estimated by fitting an exponential regression of DNAm load on the age of animals (years). DNAm status was included as a variable to predict the age of animals using five Bayesian regression models: BRR^29^, BayesA^29^, BayesB^29^, BayesCπ^36^, and Bayesian LASSO (BLASSO)^37^, as follow:

$${\varvec{y}}={\varvec{X}}{\varvec{b}}+\sum_{i=1}^{k}{{\varvec{m}}}_{{\varvec{i}}}{\boldsymbol{\alpha }}_{{\varvec{i}}}{{\varvec{\updelta}}}_{{\varvec{i}}}+{\varvec{e}}$$

where ${\varvec{X}}$ and ${\varvec{e}}$ have been previously described; ${\varvec{y}}$ corresponds to the vector of ages in years; ${\varvec{b}}$ corresponds to the vector of fixed effects, including the intercept and the linear covariates for each breed; ${{\varvec{m}}}_{{\varvec{i}}}$ is the vector of DNAm status for site *i* (coded as 0, 1, and 2); ${\boldsymbol{\alpha }}_{{\varvec{i}}}$ i is the effect of DNAm status for site *i* for each of* k* sites; ${{\varvec{\updelta}}}_{{\varvec{i}}}$ is an indicator whether DNAm status site *i* was included (${{\varvec{\updelta}}}_{{\varvec{i}}}$ = 1) or excluded (${{\varvec{\updelta}}}_{{\varvec{i}}}$ = 0) from the model for a given iteration of gibbs sampling (BayesRR and BayesA, ${{\varvec{\updelta}}}_{{\varvec{i}}}$ = 1). In BayesRR and BayesCπ, the effect of DNAm status is assumed to follow a normal distribution. In BayesA and BayesB, the effect of DNAm status is assumed to follow a *t*-distribution with site-specific variances. In Bayes Lasso, the effect of DNAm status is assumed to follow a double-exponential distribution.

Gibbs sampling was used to sample a posterior parameter distribution with a chain length of 20,000 iterations, burn-in of 2,000 samples, and a thinning interval of 100. Bootstrapping (*n* = 400) was used to evaluate the performance of the models with 102 and 34 individuals as training and validation populations, respectively. The performance of the models was evaluated based on the correlation between true and predicted age, mean square error, and slope of the regression of predicted age on true age. Analyses were performed in BGLR package^35^ in R software.

## Conclusion

Estimates of heritability suggest generational inheritance of DNAm load in the genome of cattle. The heritability estimates of each DNA site were moderate and well distributed across different functional regions of the genome, locations related to CpG islands, and chromosomes. The variation of heritability across the genome indicates the possibility of using DNAm as an additional source of information to improve phenotypic prediction. However, the effect of age on DNAm differed across the regions of the genome. The promoter and 5′ UTR were initially hypomethylated, and DNAm levels increased with age, while there was initial hypermethylation in the exonic, intronic, 3′ UTR, and intergenic regions followed by a decrease in DNAm levels with age. The high prediction accuracy of true age based on DNAm indicates a potential use of DNAm to predict chronological age in beef cattle. Overall, these findings indicate DNAm profiles are determined by both genetics and environmental influences such as age in beef cattle.

## Supplementary Information

Supplementary Information 1.

Supplementary Information 2.

## Data Availability

The methylation data will be released to Gene Expression Omnibus as part of the data release from the Mammalian Methylation Array Consortium. The mammalian methylation array (HorvathMammalMethylChip40) is registered at the NCBI Gene Expression Omnibus as platform GPL28271. The mammalian methylation array is distributed by the non profit Epigenetic Clock Development Foundation https://clockfoundation.org/.
