# Supplementary figures and images for: DNA methylation profile in beef cattle is influenced by additive genetics and age
Source: Sci Rep. 2022 Jul 14;12:12016. doi: 10.1038/s41598-022-16350-9 (PMC9283455; doi:10.1038/s41598-022-16350-9)

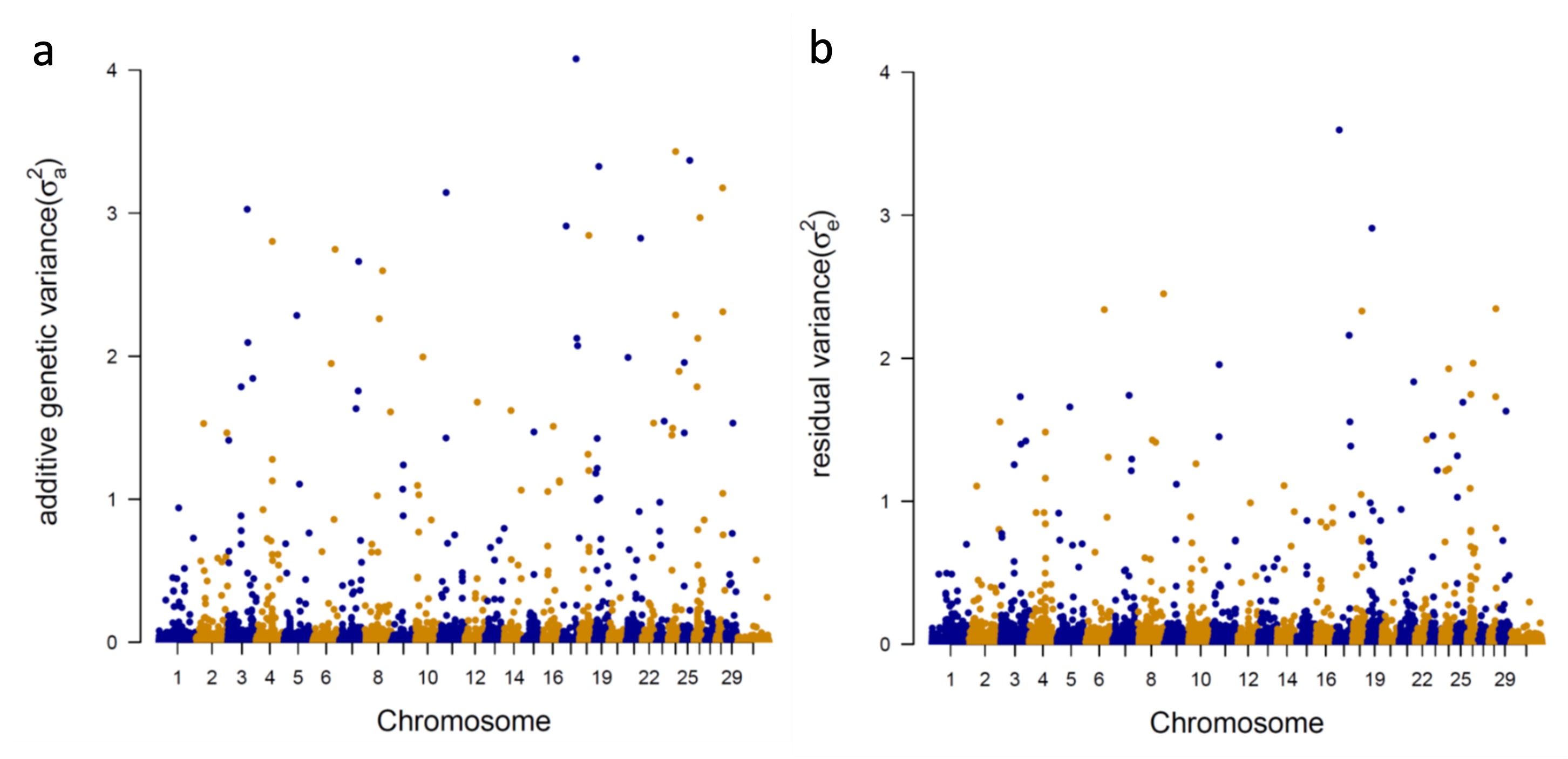

Supplement: Supplementary file 1 — Supplementary Information 1. [file 41598_2022_16350_MOESM1_ESM.jpg]

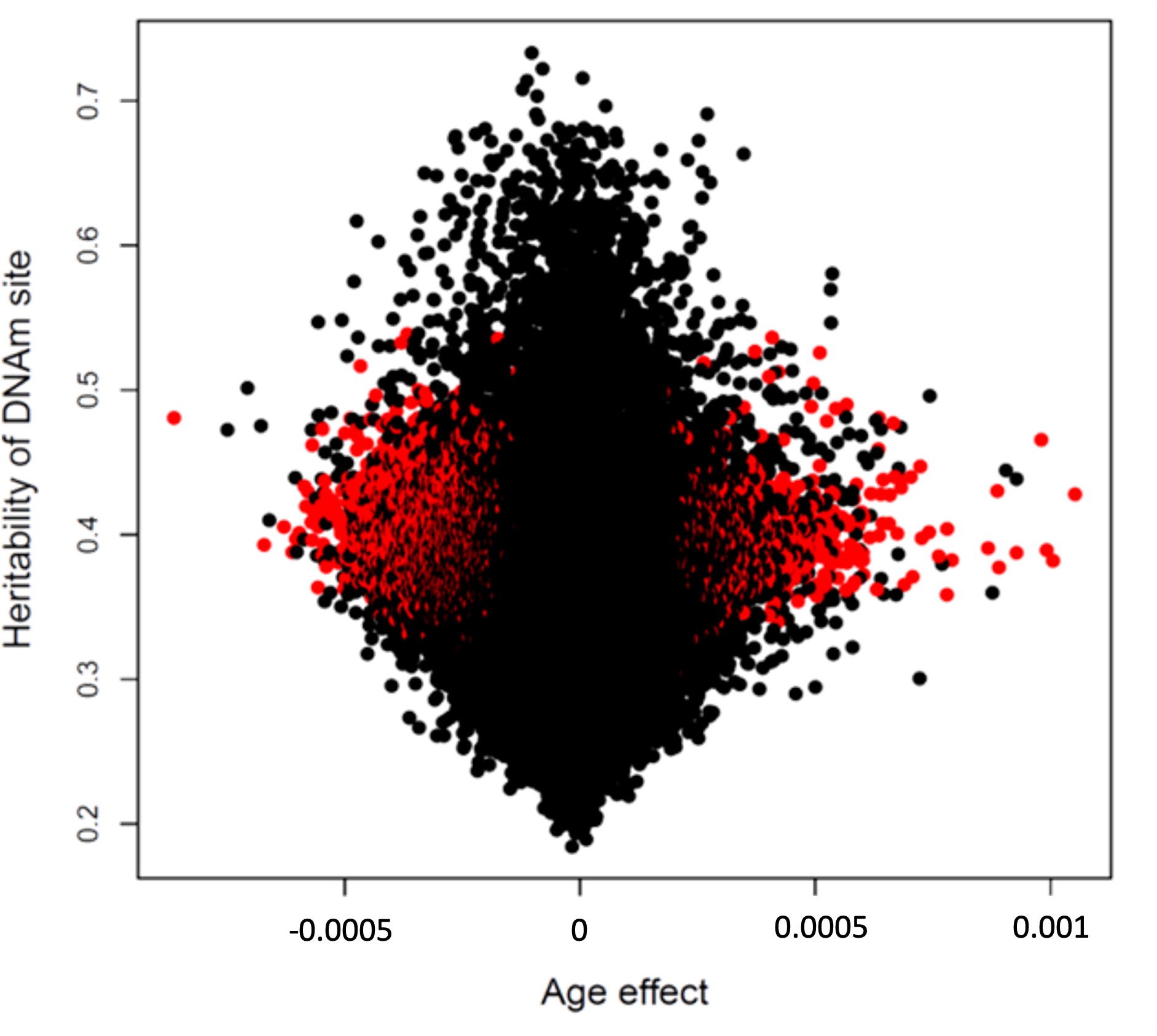

Supplement: Supplementary file 2 — Supplementary Information 2. [file 41598_2022_16350_MOESM2_ESM.jpg]
